# AagingBase: a comprehensive database of anti-aging peptides

**DOI:** 10.1093/database/baae016

**Published:** 2024-03-12

**Authors:** Kunjulakshmi R, Ambuj Kumar, Keerthana Vinod Kumar, Avik Sengupta, Kavita Kundal, Simran Sharma, Ankita Pawar, Pithani Sai Krishna, Mohammad Alfatah, Sandipan Ray, Bhavana Tiwari, Rahul Kumar

**Affiliations:** Department of Biotechnology, Indian Institute of Technology Hyderabad, Kandi, Telangana 502284, India; Department of Biological Sciences, Indian Institute of Science Education and Research, Berhampur, Odisha 760010, India; Department of Biotechnology, Indian Institute of Technology Hyderabad, Kandi, Telangana 502284, India; Department of Biotechnology, Indian Institute of Technology Hyderabad, Kandi, Telangana 502284, India; Department of Biotechnology, Indian Institute of Technology Hyderabad, Kandi, Telangana 502284, India; Department of Biotechnology, Indian Institute of Technology Hyderabad, Kandi, Telangana 502284, India; Department of Biotechnology, Indian Institute of Technology Hyderabad, Kandi, Telangana 502284, India; School of Biotechnology, Amrita Vishwa Vidyapeetham, Amritapuri, Clappana P.O., Kollam, Kerala 690525, India; School of Biotechnology, Amrita Vishwa Vidyapeetham, Amritapuri, Clappana P.O., Kollam, Kerala 690525, India; Bioinformatics Institute (BII), Agency for Science, Technology and Research (A*STAR), 30 Biopolis Street, Matrix #07-01, Singapore 138671, Republic of Singapore; Department of Biotechnology, Indian Institute of Technology Hyderabad, Kandi, Telangana 502284, India; Department of Biological Sciences, Indian Institute of Science Education and Research, Berhampur, Odisha 760010, India; Department of Biotechnology, Indian Institute of Technology Hyderabad, Kandi, Telangana 502284, India

## Abstract

The process of aging is an intrinsic and inevitable aspect of life that impacts every living organism. As biotechnological advancements continue to shape our understanding of medicine, peptide therapeutics have emerged as a promising strategy for anti-aging interventions. This is primarily due to their favorable attributes, such as low immunogenicity and cost-effective production. Peptide-based treatments have garnered widespread acceptance and interest in aging research, particularly in the context of age-related therapies. To effectively develop anti-aging treatments, a comprehensive understanding of the physicochemical characteristics of anti-aging peptides is essential. Factors such as amino acid composition, instability index, hydrophobic areas and other relevant properties significantly determine their efficacy as potential therapeutic agents. Consequently, the creation of ‘AagingBase’, a comprehensive database for anti-aging peptides, aims to facilitate research on aging by leveraging the potential of peptide therapies. AagingBase houses experimentally validated 282 anti-aging peptides collected from 54 research articles and 236 patents. Employing state-of-the-art computational techniques, the acquired sequences have undergone rigorous physicochemical calculations. Furthermore, AagingBase presents users with various informative analyses highlighting atomic compositions, secondary structure fractions, tertiary structure, amino acid compositions and frequencies. The database also offers advanced search and filtering options and similarity search, thereby aiding researchers in understanding their biological functions. Hence, the database enables efficient identification and prioritization of potential peptide candidates in geriatric medicine and holds immense potential for advancing geriatric medicine research and innovations. AagingBase can be accessed without any restriction.

**Database URL**: https://project.iith.ac.in/cgntlab/aagingbase/

## Introduction

Aging is a natural phenomenon experienced by most living organisms. As organisms progress through time, they inevitably undergo various behavioral, physiological, metabolic and structural changes indicating aging (1). These changes can manifest in several ways, such as the loss of muscle mass, compromised immune reactions, diminished skin elasticity and the emergence of fine lines and wrinkles (2–4). Moreover, as aging progresses, individuals become more susceptible to various diseases and health complications. Consequently, achieving the title of ‘aged successfully’ refers to the ability of an individual to effectively evade and mitigate the manifestation of age-associated vulnerabilities and adversities. The Global Burden of Disease 2017 study has highlighted the most prevalent causes of age-related mortality (5–7). Among these causes are cancer, cardiovascular diseases, neurodegenerative disorders and metabolic dysfunctions. These conditions not only contribute significantly to the mortality rates among the elderly population but also pose substantial challenges to healthcare systems worldwide (7–11).

A wide array of anti-aging strategies has been developed to address the complexities associated with aging. These strategies primarily focus on mitigating cellular senescence and genomic instability, tackling mitochondrial damage, rejuvenating exhausted stem cells, enhancing proteostasis, activating epigenetic mechanisms, countering telomere shortening, modulating nutrient sensing pathways, combating dysbiosis, reducing chronic inflammation, restoring proper intercellular communications, dietary modifications and attenuation of circadian and sleep disruptions (9–11). Researchers and healthcare professionals strive to develop effective interventions that promote healthy aging and extend human health spans by comprehensively addressing these various facets of aging (12, 13).

Anti-aging peptides, a class of biomolecules, have garnered considerable attention as a potential therapeutic strategy for enhancing a healthy lifespan by reducing the adverse health consequences observed in the elderly population (14, 15). Their rising popularity can be attributed to several advantages, including their immune system evasion, high precision, cost-effectiveness, simplicity of administration and safe usage (16). Compared to larger biologics, peptides possess compact sizes and exceptional membrane permeability, making them an attractive option for therapeutic interventions (17). Peptide amide bonds are easily degraded because of their significant vulnerability to enzyme hydrolysis (18, 19). Additionally, peptides’ unfavorable structures contribute to their decreased stability (20). Due to these innate qualities, peptides are prone to quick removal from the system (16). These factors can impact the efficacy and duration of their therapeutic effects.

Despite these drawbacks, anti-aging peptides offer promising mechanisms to address age-related distress. Anti-aging peptides can address age-related distress by getting involved in cell–cell communications, controlling the activity of enzymes such as collagenase, helping in the easy transportation and delivery of biomolecules and inhibiting the production of neurotransmitters such as acetylcholine (21–23). For instance, Cu-GHK is a naturally occurring peptide that is predominant in the human body and regenerates tissue and stimulates collagen formation (24). Also, Argireline is a synthetic peptide inspired by *Botulinum* toxin that exerts an anti-wrinkle effect and is popular in cosmeceuticals (25). Humanin and MOTSc are peptides derived from mitochondria that can improve mitochondrial function and biogenesis, typically declining with age (26, 27).

Furthermore, proteins such as growth differentiation factor 11 have gained prominence in anti-aging therapies due to their capacity to protect keratinocytes by activating the PI3K-AKT pathway (28). The aforementioned peptides and proteins are commonly employed to combat oxidative stress. Defensin is another protein that can help maintain a healthy microbial balance, thereby supporting the weakened immune systems of the elderly (29).

Taken together, peptide therapy has gained considerable popularity in treating aging signs. Consequently, it is necessary to adequately understand the current use of anti-aging peptides and their properties in anti-aging research and the development of related therapeutic strategies. To facilitate this process, a compilation of anti-aging peptides under one roof in a user-friendly database will be helpful. To the best of our knowledge, such a database has yet to be reported. Hence, the study aims to collect all reported anti-aging peptides and their associated information into a comprehensive database, AagingBase. We anticipate that AagingBase will be an indispensable resource for the research community working in aging, senescence, and geriatric medicine. AagingBase is available at https://project.iith.ac.in/cgntlab/aagingbase.

## Materials and methods

### Data curation and compilation

Anti-aging peptides in AagingBase were obtained from the research articles, and patents searched in PubMed and Lens databases, respectively (30, 31). In PubMed, research articles published in the time frame of 2010 to 2022 were searched using the keywords ‘anti-aging peptides’, ‘anti aging peptides’, ‘anti-ageing peptides’ and ‘anti ageing peptides’. In the Lens database, patent-related peptides were searched from 2018 to 2022 using the keywords ‘(anti-aging peptide) OR (antiaging peptide) OR (anti-ageing peptide) OR (antiageing peptide)’ with the following filters ‘have full text, sequence type: amino acid, stemness: disabled, have sequence listing, have granted patent, document type: granted patent, legal status: active’. In PubMed search, 1011 research articles were obtained, and the Lens database search yielded 4384 patents with the aforementioned keywords. According to the inclusion and exclusion criteria adopted in this study (mentioned later), 54 research articles and 236 patents were considered relevant and subsequently included in the AagingBase. Subsequently, the information from these research articles and patents was processed to gather primary and secondary data concerning anti-aging peptides.

### Inclusion and exclusion criteria

We would consider peptides with anti-aging properties only if they were experimentally validated with specific experiments targeting key indicators of anti-aging properties, i.e. DPPH (2,2-diphenyl-1-picrylhydrazyl) radical scavenging activity and the utilization of the TUNEL (terminal deoxynucleotidyl transferase dUTP nick end labeling) assay to examine genomic stability, the antioxidant enzyme activity assay that addresses cellular senescence, the mitochondrial DNA check to address mitochondrial damage, etc. (32–34). Additionally, research articles focused on enhancing the Sirtuin signaling pathway (e.g. *SIRT1* expression) and embryonic development markers (e.g. *SOX2* and *POU5F1*) were looked at (35–37). Furthermore, articles with behavioral studies examining interventions designed to improve cognitive abilities, which tend to decline with age, were sought (38–41). The availability of amino acid sequence of the geroprotective peptides of relevance was also a significant requisite in our search. Therefore, we restricted our search to the research articles and patents where peptide sequences were available.

### Database organization

#### Primary data

To obtain experimental information regarding anti-aging peptides, the filtered research articles and patents were thoroughly evaluated to acquire primary data, which include the (i) reported names of anti-aging peptides, (ii) sequence: the amino acid sequence of the anti-aging peptide that was collected, (iii) PubMed/Patent ID: the research article or patent from which the data were extracted, (iv) year of publication/patent granted, (v) origin: specifies if an anti-aging peptide is synthetic or natural in origin, (vi) source: source of naturally occurring anti-aging peptide, (vii) experimental type: the experimental design, such as *in vitro, in vivo*, *ex vivo*, etc., (viii) cell lines/animal model: the type of model system used, (ix) intervention: the method of administration of the anti-aging peptide to the model system, (x) patient group involved: the group of patients participating in the randomized clinical trial, (xi) methods: experimental methods conducted to validate the anti-aging properties, (xii) conditions addressed: the type of malady against which anti-aging peptide therapy was administered and (xiii) mechanism of action: the observed alterations resulting from the use of anti-aging peptides in the model system. Each anti-aging peptide was assigned an identification number starting with AAPeptide (Anti-Aging Peptide) as the primary key.

#### Secondary data

AagingBase displays secondary data derived from the amino acid sequence of anti-aging peptides. Secondary data are derived from amino acid sequence analysis of anti-aging peptides. Estimates are computed for atomic compositions, ProtScale values, amino acid compositions and frequencies, and secondary structure proportions using Pfeatures and Bio.SeqUtils.ProtParam module of Biopython (42, 43). Using Bio.SeqUtils.ProtParam, eight features such as molecular weight, instability index, Grand Average of Hydropathy, ProtScale values, etc., were calculated. Thirty characteristics, such as the composition of negatively charged residues and positively charged residues, were computed using Pfeature. In total, 38 physicochemical features were calculated (Supplementary Table S1). The tertiary structures of the anti-aging peptides were also predicted using *in silico* tools. PepStrMod was used to predict the structures of peptides of length within the range of 7–25 amino acids, while Iterative Threading ASSEmbly Refinement was used to predict the tertiary structures of anti-aging peptides with lengths >25 amino acids (44, 45). In total, the tertiary structures of 153 anti-aging peptides were predicted successfully. The tertiary structure of peptides with lengths of <7 amino acids is relatively difficult (46); therefore, their tertiary structures were not included in AagingBase.

### Comparison with UniProtKB peptides

To better understand the amino acid composition of anti-aging peptides, a comparative study was conducted between the AagingBase peptides and other biological peptides obtained from UniProtKB (47). We obtained peptides from UniProtKB with <80 amino acid residues (excluding peptides with unnatural amino acids). The amino acid compositions of these selected UniProtKB peptides were calculated using Pfeatures (43). Then, we randomly selected 282 UniProtKB peptides and compared the composition of each amino acid with their respective composition in AagingBase peptides using a T-test (ttest_ind library was used from scipy.stats) (48), and this process was iterated 100 times. *P*-value < 0.01 was used as a cut-off to identify amino acids with significantly different amino acid compositions between UniProKB and AagingBase peptides.

### Motif discovery

The MEME Suite 5.5.3 Motif Discovery Tool, the Multiple Em for Motif Elicitation (MEME) algorithm, was employed to identify anti-aging motifs (49). Command line-based MEME was deployed on anti-aging peptides with length ≥8 amino acids (*n* = 140) to search for the top five motifs. The motifs were required to have a minimum width of six residues and a maximum width of 50 residues.

### Database architecture

AagingBase has been built on an Ubuntu-based machine’s Apache HTTP Server (v2.4.54). The front-end interface has been developed using HTML5, CSS3, PHP5 and JavaScript. Back-end development uses MySQL/10.4.25-MariaDB to fetch the data. It is compatible with Chrome and Firefox web browsers and is designed for mobile, tablet and desktop.

## Results

### Database statistics and analysis

**Figure 1.**
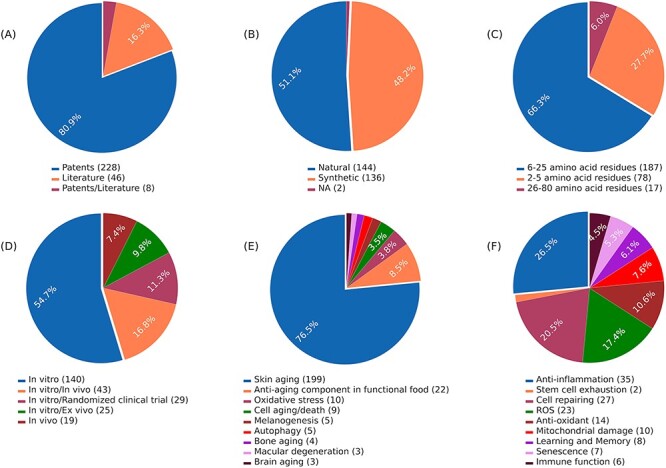
Data statistics of the AagingBase: (A) number of research articles and patents referred to in the AagingBase, (B) distribution of natural and synthetic peptides. It is the origin of the peptide based on its source, (C) length distribution of the peptides of the AagingBase, (D) experiment types performed using the peptides to validate the anti-aging property, (E) the maladies addressed using the peptides, (F) anti-aging bioactivity exhibited by the peptides.

Note: Due to complexities in classification, the total count in subfigures D, E and F may not reach 282 peptides as not all classification cases are depicted in these subplots. It is important to note that some peptides exhibit multiple or overlapping characteristics, contributing to the variability in the depicted counts.

**Figure 2.**
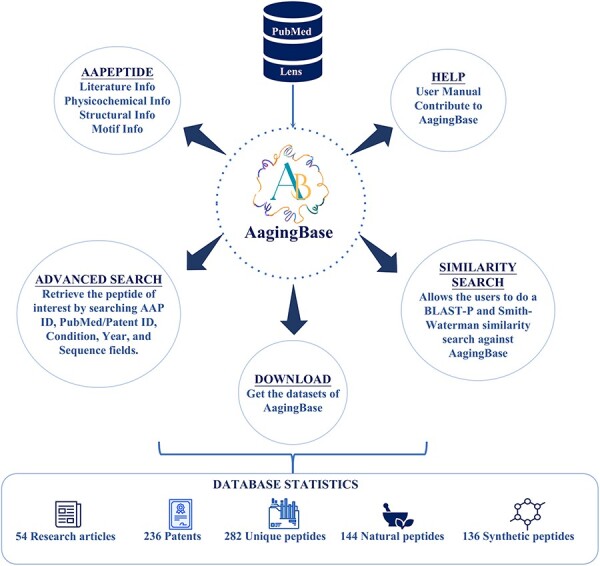
The overall architecture of the AagingBase.

AagingBase houses 282 experimentally validated unique anti-aging peptides collected from 46 research articles and 228 patents (Figure 1A). Of the 282 anti-aging peptides, 144 are natural, 136 are synthetic peptides, and 2 peptides have unknown origins (Figure 1B). In the length-wise category, most of the peptides (*n* = 187) were 6–25 amino acids long (Figure 1C). Of the 282 anti-aging peptides, 140 have been validated using *in vitro* studies (Figure 1D). Additionally, we have classified the anti-aging peptides based on the aging condition addressed and the bioactivity exhibited by the anti-aging peptides (Figure 1E and F). Skin aging was the most studied condition, where 199 peptides were studied for skin aging (Figure 1E). This highlights the applicability of anti-aging peptides in the cosmetic industry (50, 51). In the bioactivity classification, peptides exhibit functions such as anti-inflammatory activity (*n* = 35), cell repairing (*n = *27), antioxidant activity (*n* = 14), enhancing learning and memory (*n* = 8), etc. (Figure 1F). It is important to note that, due to complexities in classification, all the classifications were not considered in Figures 1D, E, and F. Figure 2 depicts the overall architecture of AagingBase.

**Figure 3.**
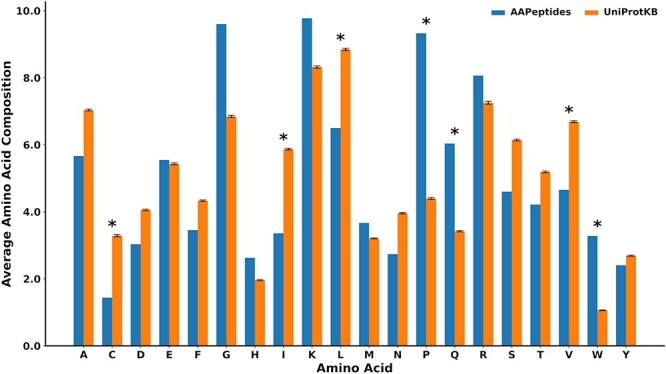
The comparison of the mean amino acid compositions between the anti-aging peptides and other biological peptides collected from UniProtKB. These are peptides with lengths of <80 amino acids, where *n* = 282 (standard deviation calculated using 100 iterations). *represents significant amino acids with *P < 0.01*

We also analyzed the amino acid composition of anti-aging peptides with respect to the peptides from UniProtKB (47). It was found that proline, tryptophan and glutamine were significantly enriched in anti-aging peptides (*P* < 0.01) (Figure 3 and Supplementary Table S2) (52, 53). This observation concurs with prior research, which has already reported the ameliorative influence of tryptophan and proline in aging (52–54). On the other hand, cysteine and branched-chain amino acids, namely isoleucine, valine and leucine, were found to be at low frequency (*P* < 0.01) in anti-aging peptides.

We also conducted the motif discovery on 140 anti-aging peptides of length 8 to 80 amino acids. The most frequently occurring motif was ‘WTWKGK’ (E-value = 7.8e − 27). Other significant motifs were ‘ALSSRLRF’ (E-value = 9.2e − 21) and ‘WVQFKIQWNQR’ (E-value = 3.7e − 14). A comprehensive summary of the discovered motifs, including their widths, occurrence frequencies and E-values, is provided in the accompanying Supplementary Table S3.

### Database web interface

AagingBase is a comprehensive database of peptides with a user-friendly interface for easy navigation (Figure 2). It offers navigation through six navigation bars, i.e. Home, AAPeptide, Advanced Search, Similarity, Help, and Team. Each peptide was assigned a unique identification number starting with AA, where AA stands for anti-aging. This identification number is a primary key linked with their respective peptide card. It displays all the primary and secondary data and graphical representations of the calculated physicochemical properties and the predicted tertiary structure.

#### AAPeptide

The AAPeptide web page has submodules: (i) literature info, (ii) physicochemical info, (iii) structural info, and (iv) motif info.

All the submodules display the data in a tabular form. The tables are sortable and have a filtering option.

- Literature info: The page displays the primary data of the anti-aging peptides. Users can access the primary data, which are reported name, PubMed/Patent ID, year, origin, experiment type, cell lines/animal model, condition addressed and bioactivity. The 282 peptides are displayed through 15 sub-pages, where each sub-page shows 20 entries in a table.
- Physicochemical info: The page showcases the secondary data of the anti-aging peptides. These are the calculated physicochemical information from the sequence of the anti-aging peptides. Twenty-six physicochemical information of the anti-aging peptides are shown (Supplementary Table S2).
- Structural info: Similar to the physicochemical page, the structural info page has secondary data, i.e. the calculated secondary structure fractions. It displays the helix, turn and sheet fractions of the anti-aging peptides.
- Motif info: The motif info page has a table showing the motif discovery results conducted. The table maintained has the graphical representation of the motifs discovered, the width of the motif, its frequency of occurrences, the corresponding E-values, and the anti-aging peptides displaying the motifs.

#### Advanced Search

The Advanced Search page allows a keyword search across the database. Users can search through the search fields: AAP ID, PubMed ID, Patent ID, condition addressed, year and sequences. The resultant page displays a table that has information on peptide length, sequence, *in vivo* stability, experiment type, model, intervention, method, mechanism of action, and PubMed ID/Patent ID.

#### Similarity

The similarity page has the submodules (i) Basic Local Alignment Search Tool for Proteins (BLAST-P) and (ii) Smith–Waterman (55, 56). Users can look for the similarity of a query peptide against the anti-aging peptides of the AagingBase.

- BLAST-P: The page offers a BLAST-P search tool with an e-value 1e − 6, allowing users to compare peptide sequences against anti-aging peptides in AagingBase. This tool compares a protein of interest to a protein database, here it is AagingBase. Users can perform a detailed exploration, uncovering significant matches and potential relationships between the query sequences and the anti-aging peptides in AagingBase.
- Smith–Waterman: The page provides users with a Smith–Waterman similarity search tool tailored to analyze peptide sequences against the anti-aging peptides within AagingBase. This tool performs a precise local sequence alignment, identifying specific similarities between sequences within the dataset. Users can perform a fine-tuned analysis, pinpointing local similarities with high accuracy to explore relationships among the provided anti-aging peptides.

It is recommended that peptides with <17 amino acids use the Smith–Waterman similarity search. The similarity search result page displays the top hits along with alignment.

#### Download

Users interested in availing the datasets of AagingBase can utilize the download page. Users can get anti-aging peptide sequences and secondary data—the Comma-Separated Value files of amino acid composition, amino acid frequency, physicochemical information and secondary structure fraction.

#### Help

The Help page has been designed to streamline user interaction. It contains the submodules (i) User Manual and (ii) Contribute.

(i) User Manual: This module contains graphical representations and descriptive content, aiding users in comprehending the functionalities and navigation within AagingBase. It also includes the terms of use and allows users to submit queries to the support team.

(ii) Contribute: Within this section, users are encouraged to submit anti-aging peptides that are currently absent from AagingBase.

## Discussion

Despite the tremendous advancements in molecular medicine in the 21st century, the field of anti-aging research has been progressing relatively slowly, primarily due to the inadequate understanding of the underpinning multidimensional physiological and metabolic complexities involved in aging and related health conditions. However, the ‘Hallmarks of Aging’’ concept provides a valuable framework for understanding the aging process and developing strategies for anti-aging investigations for extending a healthy lifespan (57). Additionally, the public’s growing interest in cosmeceutical products targeting skin aging signifies the demand for solutions that combat the detrimental health consequences of aging (58). Hence, considering the complexity of aging and the growing need to establish a ‘healthy aging’, a comprehensive approach to developing anti-aging interventions using biotechnological advancements is essential.

While finding an ‘elixir’ for aging remains elusive and captivating, AagingBase represents a modest step toward compiling peptides used in aging and age-related conditions. It allows the proper channeling of advancing biotechnological methods for peptide therapeutics for their effectiveness in anti-aging interventions. The resources of AagingBase can be coherently used with existing anti-aging therapeutics such as anti-aging drugs, calorie and dietary restrictions, senolytics, rejuvenation strategies such as cellular reprogramming and replenishing stem cell niches (59).

AagingBase serves as a valuable resource for researchers, scientists and clinicians, offering several key functionalities. Firstly, it facilitates the verification of the reported anti-aging properties of specific peptides, enabling users to explore the existing literature. Secondly, it provides access to information on the model systems and experimental methods employed to validate these anti-aging properties, including insights into their underlying action mechanisms. The clinical trial data on the patient group involved allow an insight into the nature and diversity of the clinical trial conducted. Additionally, AagingBase aids in comprehending the physicochemical and structural characteristics of anti-aging peptides associated with anti-aging effects, assisting in further investigations. Lastly, it offers valuable insights into amino acid composition and frequencies, which can be leveraged for the future design of novel, more effective geroprotective peptides.

AagingBase also offers a similarity search feature that leverages the BLAST-P and Smith–Waterman algorithms. This functionality is pivotal in aging research, empowering users to explore and manipulate peptide sequences. Researchers can effectively identify sequence similarities and relationships by employing these algorithms, facilitating in-depth analysis and further advancements in aging-related investigations.

AagingBase is expected to empower researchers to achieve desirable outcomes by facilitating the manipulation and exploitation of peptide properties for anti-aging therapies. Moreover, it enables an efficient experimental setup for designing novel therapeutic strategies by providing access to comprehensive information on previous studies, thereby minimizing time and resource expenditures. In our analysis, we found significant enrichment of proline and tryptophan in anti-aging peptides, which resonates with prior studies showing the anti-aging effect of these amino acids (52–54, 59, 60). Additionally, our identified anti-aging motifs consistently include tryptophan, which is in coherence with the previous findings showing the anti-aging role of tryptophan. Conversely, our analysis also revealed reduced levels of cysteine and branched-chain amino acids in anti-aging peptides, supporting earlier indications that manipulating these amino acids could serve as an intervention to promote healthy aging and enhance health span (61, 62). However, it is also important to acknowledge the limitations of the anti-aging peptides because of their toxicity and limited experimental validation. Even though the entries are restricted to experimentally validated anti-aging peptides, thorough research on them is essential before proceeding to their further exploitation for clinical use.

In summary, to the best of our knowledge, AagingBase reported here is the first extensive collection of anti-aging peptides. It is a comprehensive platform that allows researchers to get relevant information about anti-aging peptides used to combat and understand aging phenomenon. Furthermore, the motifs discovered in the peptides can offer valuable insights into their potential functional and structural significance. Hence, an inclusive approach to using peptide therapeutics, other anti-aging interventions and lifestyle habits will be a game-changer in combating the detrimental effects of the aging process. This database will interest many neuroscience and pharmacology disciplines, including anti-aging and longevity research, geriatric medicine, peptide drug discovery, neuropharmacology, clinical neuroscience, neurology of aging and neurophysiology.

## Supplementary Material

baae016_Supp

## Data Availability

Datasets used and generated in this study are available at https://project.iith.ac.in/cgntlab/aagingbase/.
