# Supplementary material for: AagingBase: a comprehensive database of anti-aging peptides
Source: Database (Oxford). 2024 Mar 12;2024:baae016. doi: 10.1093/database/baae016 (PMC10930205; doi:10.1093/database/baae016)
Supplement: baae016_Supp [file baae016_supp.zip › suppl_data/AagingBase_Supplementay_file.docx]

**AagingBase: A Comprehensive Database of Anti-aging Peptides**

**Kunjulakshmi R^1,2^, Ambuj Kumar^1^, Keerthana Vinod Kumar^1^, Avik Sengupta^1^, Kavita Kundal^1^, Simran Sharma^1^, Ankita Pawar^3^, Pithani Sai Krishna^3^, Mohammad Alfatah^4^, Sandipan Ray^1^, Bhavana Tiwari^2^, Rahul Kumar^1*^**

^1^Department of Biotechnology, Indian Institute of Technology Hyderabad, Kandi, Telangana 502284, India

^2^Department of Biological Sciences, Indian Institute of Science Education and Research, Berhampur, Odisha 760010, India

^3^School of Biotechnology, Amrita Vishwa Vidyapeetham, Amritapuri, Clappana P.O, Kollam, Kerala, 690525, India

^4^Bioinformatics Institute (BII), Agency for Science, Technology and Research (A*STAR), 30 Biopolis Street, Matrix #07-01, Singapore 138671, Republic of Singapore

*Correspondence: [rahulk@bt.iith.ac.in](mailto:rahulk@bt.iith.ac.in)

**Supplementary Table 1:** 38 physicochemical properties calculated and deployed in AagingBase.

| **Sl. No.** | **Physicochemical Properties*** |
| --- | --- |
|  | Peptide length |
|  | Molecular weight |
|  | Aromaticity |
|  | GRAVY(Grand Average of Hydropathy) |
|  | Oxidised Molecular extinction coefficient |
|  | Reduced Molecular extinction coefficient |
|  | Instability index |
|  | ProtScale Values |
|  | PCP_PC → Composition of positively charged residues |
|  | PCP_NC → Composition of positively charged residues |
|  | PCP_NE → Composition of neutral charged residues |
|  | PCP_PO → Composition of polar residues |
|  | PCP_NP → Composition of non-polar residues |
|  | PCP_AL → Composition of residues having aliphatic side chain |
|  | PCP_CY → Composition of residues having cyclic side chain |
|  | PCP_AR → Composition of aromatic residues |
|  | PCP_AC → Composition of acidic residues |
|  | PCP_BS → Composition of basic residues |
|  | PCP_NE_ph → Composition of neutral residues based on pH |
|  | PCP_HB → Composition of hydrophobic residues |
|  | PCP_HL → Composition of hydrophilic residues |
|  | PCP_NT → Composition of neutral residues |
|  | PCP_HX → Composition of hydroxylic residues |
|  | PCP_SC → Composition of residues having sulphur content |
|  | PCP_SS_HE → Composition of residue in secondary structure (Helix) |
|  | PCP_SS_ST → Composition of residue in secondary structure (Strands) |
|  | PCP_SS_CO → Composition of residue in secondary structure (Coil) |
|  | PCP_SA_BU → Composition of residue in solvent accessibility (Buried) |
|  | PCP_SA_EX → Composition of residue in solvent accessibility (Exposed) |
|  | PCP_SA_IN → Composition of residue in solvent accessibility (Intermediate) |
|  | PCP_TN → Composition of tiny residues |
|  | PCP_SM → Composition of small residues |
|  | PCP_LR → Composition of large residues |
|  | PCP_Z1 → Composition of residues having Z1 advanced Physico-chemical properties |
|  | PCP_Z2 → Composition of residues having Z2 advanced Physico-chemical properties |
|  | PCP_Z3 → Composition of residues having Z3 advanced Physico-chemical properties |
|  | PCP_Z4 → Composition of residues having Z4 advanced Physico-chemical properties |
|  | PCP_Z5 → Composition of residues having Z5 advanced Physico-chemical properties |

*Serial numbers 1 to 8 are calculated using Bio.SeqUtils.ProtParam and the rest are calculated using Pfeatures.

**Supplementary Table 2:** Result of the T-test conducted to compare amino acid composition between anti-aging peptides (*n = 282*) and peptides from UniProtKB (*n = 282*)

| **Sl no** | **Amino Acid** | **t-statistic** | **p-value** |
| --- | --- | --- | --- |
|  | P* | 5.55 | 4.31E-08 |
|  | W* | 4.40 | 1.27E-05 |
|  | C* | -4.39 | 1.34E-05 |
|  | I* | -4.35 | 1.60E-05 |
|  | V* | -3.82 | 51E-04 |
|  | Q* | 3.58 | 3.67E-04 |
|  | L* | -2.99 | 2.94E-03 |
|  | G | 2.53 | 0.011 |
|  | S | -2.39 | 0.012 |
|  | D | -2.25 | 0.025 |
|  | N | -2.21 | 0.028 |
|  | A | -1.56 | 0.118 |
|  | T | -1.34 | 0.180 |
|  | R | 1.26 | 0.209 |
|  | K | 1.17 | 0.244 |
|  | H | 0.96 | 0.339 |
|  | M | 0.85 | 0.398 |
|  | F | -0.76 | 0.445 |
|  | E | -0.65 | 0.518 |
|  | Y | 0.64 | 0.524 |

**Supplementary Table 3:** Top five anti-aging motifs identified using MEME suite.

| **Motifs** | **Width** | **Frequency** | **E-value** |
| --- | --- | --- | --- |
| 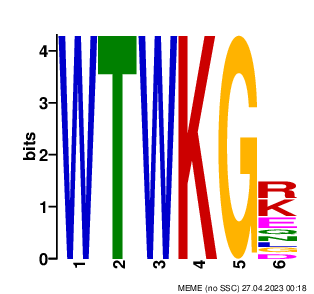 | 6 | 13 | 7.8e-027 |
| 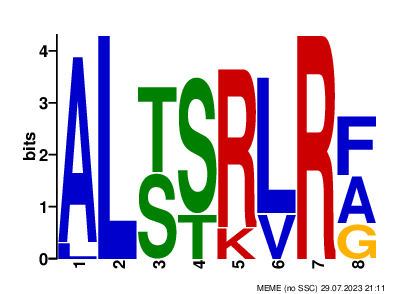 | 8 | 12 | 9.2e-021 |
| 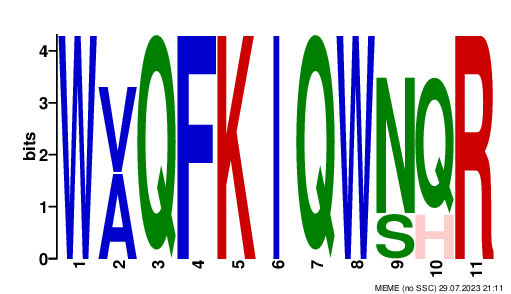 | 11 | 4 | 3.7e-014 |
| 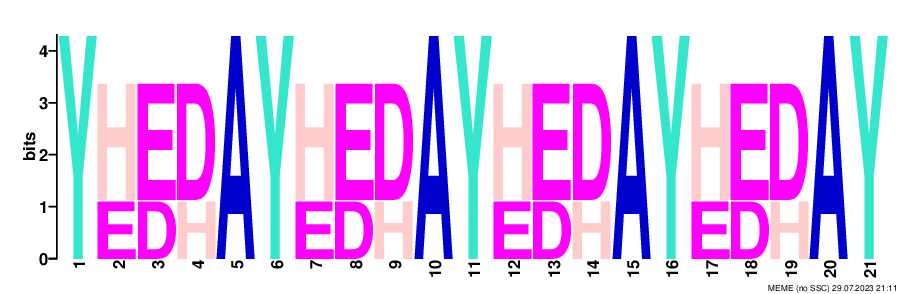 | 21 | 3 | 1.7e-013 |
| 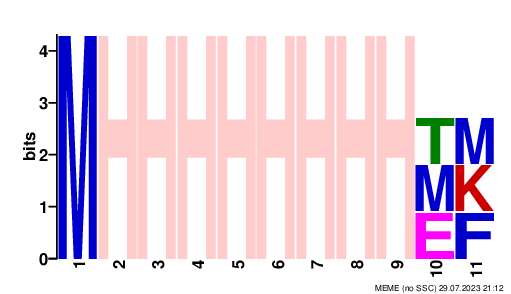 | 11 | 3 | 1.0e-011 |
